# Snapshot of Fall Prevention in Patients Referred to a Neurorehabilitation Unit: A Feasibility Study on the Use of an Airbag Device

**DOI:** 10.3390/s24196272

**Published:** 2024-09-27

**Authors:** Laura Comini, Adriana Olivares, Lucia Marchina, Adrian Suruniuc, Fabio Vanoglio, Gian Pietro Bonometti, Alberto Luisa, Giacomo Corica

**Affiliations:** 1 Scientific Direction of the Institute of Lumezzane, Istituti Clinici Scientifici Maugeri IRCCS, 25065 Lumezzane, Italy; adriana.olivares@icsmaugeri.it; 2 Neurological Rehabilitation of the Institute of Lumezzane, Istituti Clinici Scientifici Maugeri IRCCS, 25065 Lumezzane, Italy; lucia.marchina@icsmaugeri.it (L.M.); fabio.vanoglio@icsmaugeri.it (F.V.); alberto.luisa@icsmaugeri.it (A.L.); 3 Neuromotor Rehabilitation of the Institute of Lumezzane, Istituti Clinici Scientifici Maugeri IRCCS, 25065 Lumezzane, Italy; adrian.suruniuc@icsmaugeri.it (A.S.); giampietro.bonometti@icsmaugeri.it (G.P.B.); 4 Health Direction of the Institute of Lumezzane, Istituti Clinici Scientifici Maugeri IRCCS, 25065 Lumezzane, Italy; giacomo.corica@icsmaugeri.it

**Keywords:** smart belt, airbag, falls, prevention, in-hospital rehabilitation

## Abstract

Active wearable devices such as protective smart belts have been proposed to reduce hip impact in the event of a fall. This study primarily evaluated the feasibility and acceptance of a specific protective belt among selected patients identified as being at risk of falling who were admitted to an ICS Maugeri Neurorehabilitation Unit from September 2022 to April 2023. According to previous institutional observations, the device was worn between the 6th and 21st days of recovery. Out of 435 admitted patients, 118 were considered eligible, but 101 declined to participate (about 50% refused to wear the belt without first trying it on; the other 50% found it too heavy or difficult to manage). Among the 17 patients who accepted (users), 9 used the belt correctly. The remaining eight patients refused to wear it after 24 h, due to discomfort. Out of 435 patients admitted, we observed at least one fall in 49 patients, of whom 5 were eligible patients; 1 was a user who had quickly refused to use the belt and fell with mild damage. Two non-eligible patients and one eligible non-user patient experienced falls resulting in hip fractures; only in the latter case could the use of the belt have limited the damage to the hip. Difficulties in recruiting patients and low acceptance of the proposed intervention present further challenges.

## 1. Introduction

The accelerated aging of the population has significantly increased the global healthcare burden [1] as people live longer. The Global Burden of Disease study reported nearly 17 million years of life lost from falls in 2017 [2]. The literature indicates that the global proportion of older adults will rise from 10% to 16%, with about one in every six individuals being 65 years or older by 2050 [3], which will be associated with the presence of multimorbidity, polypharmacy, and frailty. All of these factors affect both functional and cognitive decline [4], notably resulting in impaired balance control, altered gait patterns, reduced mobility, and falls [5]. In terms of falls, there are differences based on the setting, with the incidence of falls being higher for older adults living in care homes or during hospital stays [6]. 

Rehabilitation centers admit older, chronic, disabled, and frail patients, a population long known to be at high/very high risk of falls [7], but studies on this setting are limited. As falls are among the most frequent adverse events in healthcare facilities and can lead to immediate and even serious long-term consequences (sometimes resulting in the death of the patient), preventing negative health outcomes from falls has a significant impact both on patients’ health and healthcare costs. It is estimated that about 14% of falls in hospitals can be classified as accidental—caused by environmental factors (e.g., slipping on a wet floor)—and 8% as unpredictable, given a patient’s physical condition (e.g., sudden disturbance of balance), but 78% of falls could be classified as predictable due to identifiable risk factors associated with the patient (e.g., disorientation and difficulty walking) [8]. Predicting and proactively preventing falls entails addressing the resultant outcomes, which contribute to increased costs for hospitals due to prolonged inpatient stays and necessary diagnostic and therapeutic procedures. Furthermore, the repercussions of falls may give rise to the onset of new disabilities or heightened morbidity for patients, potentially leading to a substantial increase in costs [9].

An analysis of existing data on fall risk recorded at the Istituti Clinici Scientifici (ICS) Maugeri IRCCS in Lumezzane (Brescia, Italy) for the years 2022 and 2023 showed that the incidence of falls was 5.8% and 2.9% per 1000 days of hospitalization for patients admitted to the Neurological Rehabilitation Unit, and 3.0% and 1.2% per 1000 days for patients admitted to the Neuromotor Rehabilitation Unit, respectively. Within these percentages, 50% of the falls resulted in patient injuries. Additionally, it was observed that the peak occurrence of falls took place between the second and third week of hospitalization, specifically during both daytime (6:00 a.m. to 4:00 p.m.) and nighttime (10:00 p.m. to 6:00 a.m.) periods.

Over the years, significant efforts have been made to develop and improve assessment tools for patients identified as “at risk of falling” [10]. These efforts aim to implement protocols to reduce risks, including personalized preventive measures such as restraints, harnesses, and floor markings. Recently, emerging technology has offered additional intervention in the form of active wearable devices, such as protective smart belts that employ the airbag principle [11,12]. These devices are designed to reduce the impact on the hip in the event of a fall and have predominantly been targeted for home use. Indeed, there is a scarcity of research demonstrating the effectiveness of wearable protectors in hospital environments, particularly in the context of neurological rehabilitation.

This study primarily sought to assess the feasibility and acceptance of intra-hospital utilization of a specific protective belt in a selected patient population, and to determine whether its continued use is justified once the patient has returned home. A secondary aim was to evaluate whether, during the study, the use of the wearable device could limit the damages caused by a fall in patients identified as at risk of falling (i.e., eligible patients), when compared to those who did not use the device (i.e., non-eligible patients).

## 2. Materials and Methods

This is a feasibility study that received approval from the Maugeri Ethical Committee (Protocol Number CE 2662, 14 September 2022). Patients signed a written informed-consent form prior to participation. 

### 2.1. Patients and Eligibility Criteria

During the period of September 2022–April 2023, patients comprising adults of both sexes who were consecutively admitted to the ICS Maugeri IRCCS in Lumezzane (Brescia, Italy), to either the Neurological or Neuromotor Rehabilitation Unit, for a period of rehabilitation were considered. Regardless of clinical diagnosis, we evaluated all patients, including those with neurological and orthopedic diseases. 

Due to an initial inventory of nine wearable belts provided to the units, using objective factors, we tried to identify which patients would benefit from wearing these belts and the order of priority for wearing them. First, patients with a recovery period of less than six days (due to death or transfer to another hospital) were excluded. Then, we considered patients who had to be excluded from enrollment in the study as a result of not meeting the eligibility criteria. These included patients who were retired, had an end-of-life condition, were confined to a bed, or were unable to walk independently. Thus, we focused on patients who were potentially at high risk of falls and could derive the most benefit from wearing a protective belt. As a risk assessment of falls using ladders is mandatory upon admission to a rehabilitation program, the Morse [13] and Tinetti POMA [14] scales were chosen to screen potentially eligible patients. Patients were defined as being at risk of falling according to Morse values ≥ 40 [13] and/or Tinetti POMA values ≤ 18 [14] at the time of admission. Moreover, in the absence of specific tests (i.e., MMSE or other tests), patients who did not have cognitive decline that could interfere with appropriate belt management were prioritized. Patients were considered cognitively oriented based on the cognitive Functional Independence Measure [15] (c-FIM, values ≥ 24) and after clinical evaluation by an ICS Maugeri nurse. If patients did not meet these criteria, they were excluded.

### 2.2. Tool

Emerging wearable technologies include smart belts designed to attenuate ground impact force in the event of a serious hip-impacting fall [16]. A Hip Guard is an airbag belt intended to safeguard the hip in the event of a fall. Hip Guards are designed for older people or people with mobility problems who are at risk of falling to prevent hip damage. The belt employs the accelerometer principle and is intended to be adjusted at the waist; it should not be worn directly on the skin. In the event of a fall being detected, the airbag inflates on the left or right hip, depending on the direction of the fall, thus providing optimal protection before the impact on the ground occurs. The measurement of the patients’ waist size determined which of the six offered sizes of the smart belt was appropriate (from XS to XXL size). In the event that none of these sizes fitted and a belt was not available, the patient was monitored by a nurse during the in-hospital stay. Falls were recorded in the institutional database.

### 2.3. Protocol

All eligible patients who provided their written informed consent (IC) to participate were included in the fall-injury reduction protocol. Based on availability, selected patients were provided with a belt of an appropriate size. These patients were invited to wear the belt for two weeks (the second and third weeks of in-hospital stay), starting on the 6th day of admission. According to the study aim outlined in the Introduction and because of the limited number of belts, this was a choice based on institutional data showing that the majority of falls happen during the central phase of in-hospital recovery. Usually, in-hospital stay at a Neuromotor or Neurological rehabilitation unit for recovery from orthopedic and neurological diseases lasts a minimum of 4-to-8 weeks.

The implementation of a smart belt for each candidate was initiated with a clinical assessment by the nurses and physiotherapists. From the very first days of admission, two ward coordinating nurses were responsible for monitoring the rating scales to determine belt assignment to the patients. On day 5, the nurses reported the patients to the physiotherapists for confirmation. On day 6, the device started to be worn, according to previous observations at our institution that patients fell mainly during the daytime. The belt could be worn from 6:00 a.m. to 10:00 p.m. to protect the patients, mainly during daily activities. The patients were also recommended to wear the belt at night, especially during bathroom use. The belt was removed during resident bathing, nighttime, or device charging. Approximately every three days, when the sensor showed a decrease in charge with a red light, the patients placed the belt on the charger on their bedside table themselves. 

### 2.4. Measures 

Anthropometric data (age, sex, weight, and body mass index) and severity of comorbidities (severity index based on the Cumulative Illness Rating Scale (CIRS)) [17] were collected. In addition, several scales used during pre- and post-rehabilitation to evaluate improvement in the patients’ motor disability were considered: the Timed Up and Go Test (TUG) [18]; the Functional Independence Scale (FIM), covering both cognitive and motor components [15]; and the Barthel Index [19].

Concerning the use of the wearable device, at the end of the rehabilitation period, the following evaluations were conducted:

1. Compliance/adherence to the use of the device by the patients, expressed as the number of hours used per day/total number of hours, and the percentage of adherence, was calculated;
2. Side effects during use, such as skin lesions, allergies to device components, pain, and discomfort in wearing, calculated as the percentage of patients with side effects;
3. Rating of device usefulness during rehabilitation activities according to a 5-point Likert scale (useless, not very useful, useful, very useful, and extremely useful);
4. Patient perception of safety according to a 5-point Likert scale (useless device, little useful, useful, very useful, and extremely useful);
5. Ease of use of the device rated by the nurses and PTs according to a 5-point Likert scale (useless, not very useful, useful, very useful, and extremely useful).

#### 2.4.1. Morse Fall Scale 

The Morse Fall Scale is a widely used tool to assess the risk of falls in patients [13]. It consists of six assessment categories: history of falls, diagnosis, intravenous therapy, use of walking aids, gait, and mental status. Each category is evaluated and assigned a score, which can help healthcare professionals identify patients at risk of falls and take appropriate preventive measures. Each evaluated criterion receives a score that varies from zero to 30 points, summing up to a risk score with the following classifications: low risk, with scores ranging from 0 to 24; moderate risk, with scores ranging from 25 to 44; and high risk, with scores ≥ 45. For the current study, we arbitrarily indicated a value ≥ 40 as a cut-off for belt use.

#### 2.4.2. Tinetti Performance-Oriented Mobility Assessment (POMA) Scale 

The Tinetti test provides a balance score (A) and a gait (B) score [14]. It uses a 3-point ordinal scale of 0, 1, and 2. Balance is scored out of 16 points, and gait is scored out of 12 points, totaling 28 points. The lower the total score on the Tinetti test, the higher the risk of falling. Scores ≤ 18 are classified as high risk, scores between 19 and 23 as moderate risk, and scores ≥ 24 as low risk of falling. For the current study, we arbitrarily indicated a value ≤ 18 as a cut-off for belt use.

#### 2.4.3. Functional Independence Measure (FIM)

The FIM consists of 18 items assessing 6 areas of function [15]. The items fall into two domains: motor (13 items) and cognitive (5 items). These domains are referred to as the Motor-FIM and the Cognitive-FIM. The motor items are based on the items of the Barthel Index.

Each item on the FIM is scored on a 7-point Likert scale, and the score indicates the amount of assistance required to perform the task listed in the item (1 = total assistance in all areas, and 7 = total independence in all areas). The total score ranges from 18 to 126 (best performance).

#### 2.4.4. Timed up and Go (TUG) Test

The TUG measures the dynamic balance and functional mobility in older adults, as well as in the population with neurological disorders [18]. The TUG is a simple test that can be performed anywhere and consists of a patient getting up from a chair from the sitting position to the bipedal position, walking three meters, turning, returning, and sitting on the chair again. The variable measured is the total time taken to complete the test, and a score measured in seconds is recorded, which is correlated with the risk of falls. A time of execution ≤ 10 s is considered normal. A score ≥ 14 s indicates a high risk of falls (worse performance) [19].

#### 2.4.5. Barthel Index (BI)

The Barthel Index for Activities of Daily Living is an ordinal scale that measures a person’s ability to complete activities of daily living (ADLs) [20]. It is composed of ten items that are scored with several points, and then a final score is calculated by summing the points recorded for each functional skill. The higher the score, the more independent the patient is in completing the measured ADLs. Total score ranges from 0 to 100 (best performance).

### 2.5. Statistics

Patient data were collected during hospital stay or extracted through a business intelligence software program developed within the ICS Maugeri network (CRUSDOT-Cruscotto Direzione Ospedale Territorio—Hospital–Home Management Dashboard) and further analyzed through R programming (Vienna, Austria, 2018) for statistical analysis. The data were expressed descriptively as number, percentage (%), and mean ± standard deviation. Evaluation of Gaussian distribution was performed using the Shapiro–Wilk test. Differences between groups (i.e., eligible vs. non-eligible; fallers vs. non-fallers) were assessed using an unpaired data test (Mann–Whitney test) for quantitative variables and Pearson’s chi-square test (applying the Monte Carlo correction in the case of low numbers) for categorical ones. Comparisons of quantitative variables between admission and discharge (paired data) were assessed using the Wilcoxon test. Statistical significance was accepted when *p*-values < 0.05.

## 3. Results

### 3.1. Time of Enrollment

The evaluation period for this study encompassed admissions for rehabilitation at the ICS Maugeri (Lumezzane, Brescia) from September 2022 to April 2023. Initially, the enrollment period was planned for six months, but it was extended to eight months due to recruitment difficulties observed in the Neurological Rehabilitation Unit starting from the third month of the study.

### 3.2. Setting 

In the original project, we planned to include only patients admitted to the Neurological Rehabilitation Unit. However, we found that most patients with neurological disorders were too compromised clinically and cognitively and were often bedridden. To increase the case numbers, we decided to also include patients admitted to the Neuromotor Rehabilitation Unit. These patients were mainly patients with orthopedic diseases after surgery.

### 3.3. Personnel

This study began during a period when repeated COVID-19 outbreaks were occurring in the ward. This led to the involvement of only two nurses, whose shifts and illnesses posed a significant challenge. Additionally, the lack of dedicated staff for the project limited our ability to give patients the necessary attention, presenting a real obstacle to the feasibility of the study.

### 3.4. Participant Identification and Eligibility

**Figure 1:**
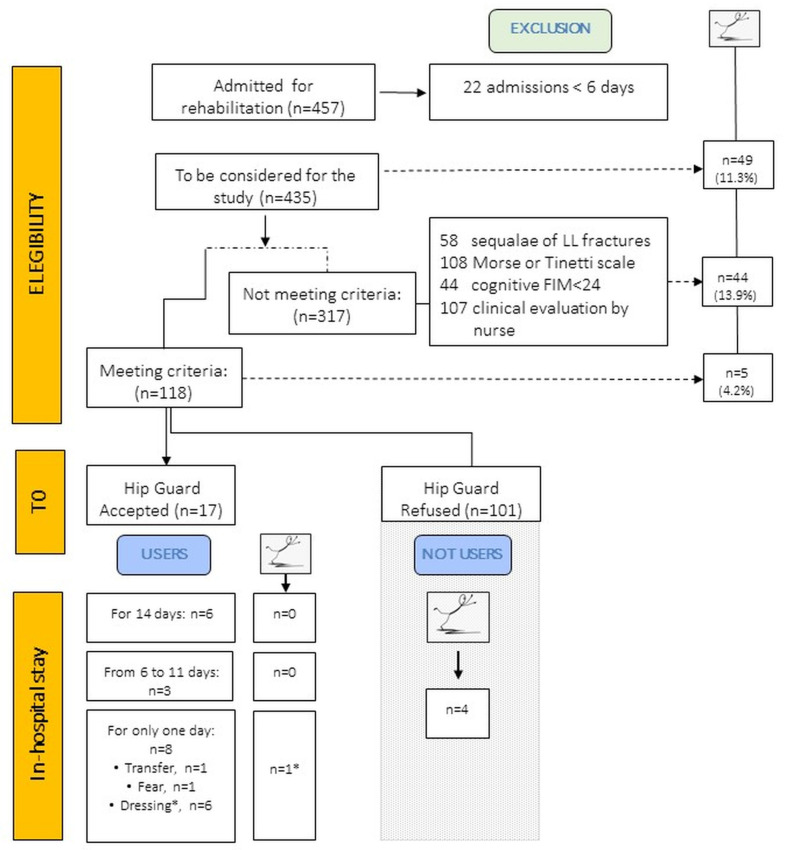
Flowchart of the study. Legend: LL, lower limb; FIM, Functional Independence Measure. * This fall occurred during the subsequent three weeks of hospitalization after the patient refused to wear the belt due to dressing problems.

Figure 1 describes the course of the study.

During the study period, we identified 457 patients admitted to rehabilitation in both units. However, only 435 patients had a recovery period longer than six days (Figure 1). After applying our specific inclusion and exclusion criteria, we identified 118 patients as potentially eligible (Figure 1 and Table 1, left side). Among these, 29 patients were admitted to the Neurological Rehabilitation Unit and 89 to the Neuromotor Rehabilitation Unit. The remaining 317 patients, including 169 from the Neurological Rehabilitation Unit and 266 from the Neuromotor Rehabilitation Unit, did not meet the study criteria (Figure 1 and Table 1, right side) and were considered “non-eligible.”

Eligible (E) patients had a significantly lower CIRS severity index and higher Barthel Index and motor FIM (both *p* < 0.01) scores; however, their scores still indicated functional limitations. They were slightly overweight and had a similar length of stay (LOS) compared to non-eligible (NE) patients (Table 1, E patients vs. NE patients).

Out of the 118 eligible patients, 101 refused to participate. About 50% of these patients refused the device outright due to excessive personal thoughts and worries, and the remaining 50% cited discomfort, mainly due to the weight of the belt or difficulty in putting it on and taking it off. These 101 patients were classified as non-users (NUs).

**Figure 2:**
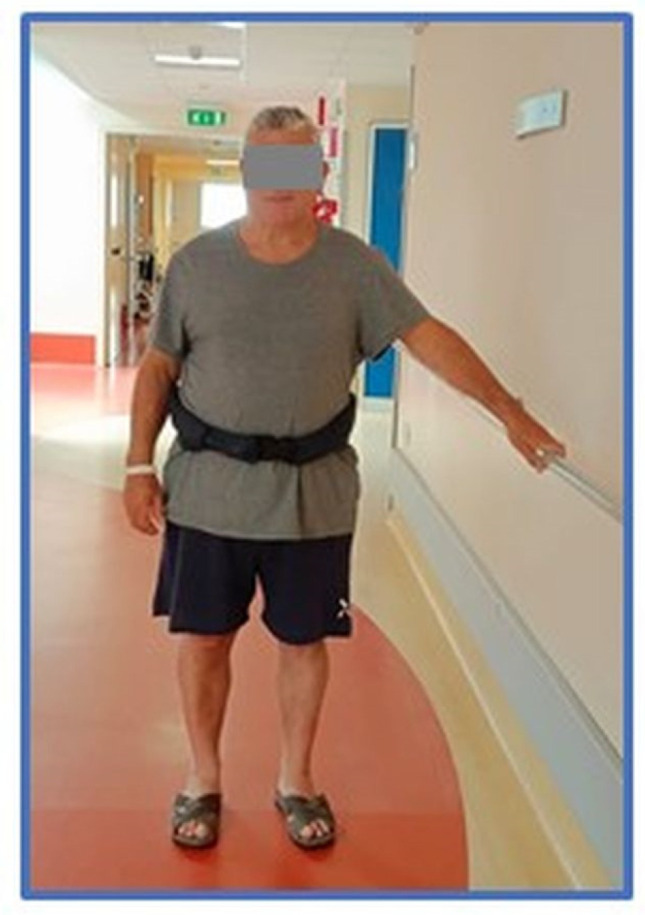
A patient wearing a Hip Guard wearable device.

Only 17 patients signed the written IC and were classified as users (Us) (Figure 2). 

Of these patients, 12 were admitted to the Neurological Rehabilitation Unit (4 due to balance problems, 4 due to walking disturbances, 2 due to amyotrophic lateral sclerosis, 1 post stroke, and 1 due to hemiparesis), and 5 to the Neuromotor Rehabilitation Unit (1 for knee replacement and 4 for hip fractures). At the time of admission, users had significantly better clinical and functional conditions (i.e., higher motor FIM and Barthel Index scores and lower TUG, all *p* < 0.05) than non-users (Table 1, Us vs. NUs in gray columns).

### 3.5. Acceptability

Among the 17 users, 53% were able to use the belt correctly: 35% (6/17) for the entire study period (14 days) and 18% (3/17) for 6-to-11 days due to discharge. Another 35% (6/17) refused the belt after 24 h due to side effects, such as discomfort, particularly for female patients. An additional patient (6%) refused to use the belt due to fear after an abnormal airbag deployment, and only one patient used the belt for a single day before being transferred to another hospital.

Compliance with the use of the device, measured as the number of hours used per day/total number of hours, was poor. Most patients left the belt on the bedside table during the day or, when using it at night, complained about difficulty reconnecting it quickly before going to the toilet.

Six questionnaires were completed by three patients who used the belt for 14 days and three who used it for 6–11 days. Two patients perceived the belt as not useful and unsafe, while the other four found it useful and rated it from safe to extremely safe. Healthcare staff (two nurses and two physiotherapists) considered the belt useful or very useful.

### 3.6. Occurrence of Falls and Injuries

At the end of the rehabilitation period, we evaluated the number of falls. A total of 75 falls were recorded, representing 4.3% per 1000 days of hospitalization (6.5% falls in the Neurological Rehabilitation Unit and 2.1% falls in the Neuromotor Rehabilitation Unit), with a maximum of eight falls per patient.

Among the entire patient population, 49 out of 435 patients fell at least once (fallers at 11.3%; Table 2, left side). These patients were primarily admitted to the Neurological Rehabilitation Unit (33/169) and Neuromotor Rehabilitation Unit (16/266) (*p* < 0.001). Patients who experienced falls were older, predominantly male, and had greater clinical disability (lower Barthel Index and motor FIM scores, and higher LOS) than those who did not fall (Table 2, left side). The major comorbidities in patients who fell included hypertension (73.5%), neurological or psychiatric diseases/neurocognitive impairment (53.1%), and cardiac diseases (51.0%). Falls most often occurred in the inpatient room (75.5%), typically in the morning (38.8%) or afternoon (38.8%), and were primarily due to slipping/stumbling (51.0%) or loss of balance/strength (38.8%) during transitions between bed, pram, or chair (65.3%). 

In 17 out of 49 fallers (37%), at least one of the related falls had slight (10 cases) or serious (7 cases) consequences, with 3 of them reporting a hip fracture (Table 3).

To evaluate whether, during the study, the use of the wearable device could limit the damages caused by a fall in patients identified as being at risk of falling (i.e., eligible patients) compared to those who did not use the device (i.e., non-eligible patients), we analyzed the number of falls and injuries in these two subgroups (Table 3). In general, falls were more frequent in non-eligible patients (13.9% vs. 4.2%, Figure 1) who were more clinically (higher CIRS and BMI) and functionally (lower FIM and Barthel Index) compromised than eligible patients (*p*-values shown in Table 1, last column). Moreover, 34% (15 out of 44) of non-eligible patients fell multiple times, with at least one fall resulting in mild or serious injuries. In particular, two cases led to serious damages caused by the removal of the restraints from the bed or wheelchair (Table 3), resulting in hip fractures. The other incidents were mainly related to abrasions.

Among the eligible patients, falls were observed in 5 out of 118 patients (4.2%, Figure 1, detailed data in Table 2, right side), with 4 patients (13.8%) in the Neurological Rehabilitation Unit and 1 patient (1.1%, *p* = 0.0127) in the Neuromotor Rehabilitation Unit (29 vs. 89 patients, respectively). There were no significant differences in functional evaluations between fallers and non-fallers, but fallers were older in age (Table 2, *p* = 0.0509). 

Of the five eligible patients who fell, one patient was included in this study as a user. However, this patient fell in the subsequent three weeks of hospitalization after refusing to wear the belt on the second day due to dressing problems. The fall, which resulted in a mild injury, happened just before discharge, when the patient experienced dizziness while getting up from a chair.

Within the eligible but non-user patients, a serious fall occurred in a patient who slowly slid out of the wheelchair, resulting in a hip injury (Table 3). 

With regard to secondary outcomes, in the two non-eligible patients with a hip fracture due to falling, the use of a smart belt would not have prevented the fall, as these patients were bedridden or unable to walk independently, and the belt was not intended for this type of patient. On the contrary, in the case of an eligible but non-user patient who used a wheelchair as a means of standing up on their own, we can conjecture that using a smart belt would have protected against hip-fracture damage.

There were also two incidents of airbag deployment that did not result in a fall on the ground with severe impact and could be considered false alarms. In one case, the airbag deployed when the patient lost balance and leaned against the bathroom wall. The patient continued to wear the belt for the remainder of the study period without further incidents. In the second case, the airbag deployed against a chair while the patient was sitting, causing such agitation that the patient refused to wear the belt thereafter.

### 3.7. Pre- and Post-Measures in 17 Users: Suggestions for Belt Wearing at Home 

To determine whether continued belt use could be justified after patients had returned home, we evaluated several outcome measures using rating scales among the user patients after rehabilitation (Table 4). 

Pre-to-post comparisons for the rating scales showed significant improvements in disability (Barthel Index: 74 vs. 90 median value, *p* < 0.0001), functional independence (motor FIM: 64 vs. 76 median value, *p* = 0.0032, and total FIM: 96 vs. 111 median scores, *p* = 0.0072), and balance (total Tinetti: 16 vs. 21 median scores, *p* = 0.0168, with Tinetti balance scores increasing from a median value of 9 to 13, *p* = 0.0334). The TUG and Tinetti gait scores did not vary significantly (Table 4). All outcome measures at discharge suggested no indication requiring belt use once the patients had returned home.

## 4. Discussion

Several assessment tools and preventive measures have been developed to address the risk of falls among patients [21]. Beck Jepsen et al. [22] and Seppala et al. [23] emphasize the importance of standardized screening tools and procedures, as well as the need to consider multifactorial causes and individual characteristics in fall-risk assessment. Zhao [24] and Ferreira [11] point to sensor-based technologies in assessing and preventing falls, while Chen et al. [12] highlight the potential of wearable airbag technology and data modeling. Together, these studies underscore the potential of innovative technologies and the importance of comprehensive, personalized approaches in mitigating the risk of falls.

Despite advancements in fall-prevention strategies, both the use of hip protectors to prevent fall-related injuries and the use of digital technology (including wearables) for detection, prevention, or management have shown low levels of agreement across guidelines. Furthermore, these guidelines [5] demonstrate a general lack of clinical applicability for fall prevention and management among adults or older individuals in various settings, including in community, acute care, and nursing homes.

### 4.1. Is the Study Intervention Feasible to Implement in Routine Clinical Practice?

During this feasibility study, the researchers encountered difficulties in recruiting and retaining in-hospital participants due to the low number of patients meeting the eligibility criteria and low patient acceptability of the device. Among the eligible patients who declined to participate, about 50% refused to wear the belt without first trying it on, while the remaining 50% agreed to try it on but subsequently found it too heavy or difficult to manage and then declined to participate further. This study highlighted challenges related to recruiting older patients and patients with neurological disorders, necessitating the researchers to involve another unit, the Neuromotor Rehabilitation Unit, to increase the study’s patient pool. Clinical complexity, advanced age, cognitive impairment, and a lack of specific guidelines were identified as the main factors limiting the implementation of the smart belt in daily practice for in-hospital patients with neurological disorders. Additionally, limited human resources further hindered project execution, as also reported by Shany [25].

There are several approaches to assessing the risk of falls in different settings. A traditional tool for the evaluation of risk of falls among inpatients is the Morse Fall Scale, a tool developed in acute hospital settings that appears to be a poor predictor of falls [26] in the rehabilitation setting. 

Most guidelines strongly recommended risk stratification using “case finding” self-reported questions, including fall history, fear of falling, and gait and balance difficulties, and reserving gait and balance testing for those who screen positive on these questions. However, indications regarding eligible candidates are lacking [5].

For these reasons, in consideration of the older patient population we admitted at our hospital and the limited number of available belts, we arbitrarily decided to use a set of scales of routinary use to assess the patients upon admission to the rehabilitation units. This was according to a systematic review published in 2018 by Park [21], pointing out that combining two assessment tools was more effective than using a single one due to the consideration of more factors. We used the Morse Fall Scale and the Tinetti scale to identify motor limitations and performed cognitive evaluation using the cognitive FIM scale to assess the risk of falls, as we, along with the existing literature, recognize this is a great limitation, particularly in our setting of patients. 

The assessment of balance and gait is recommended in 13 out of 15 guidelines [5] by means of the Timed Up and Go (TUG) Test [18], the Berg Balance Scale [27], and the Tinetti Performance-Oriented Mobility Assessment Tool [14], with the TUG being the most recommended test, appearing in 6 out of the 15 guidelines [5]. However, the predictive value of these scales remains controversial [28,29].

The use of the Functional Independence Measure (FIM) scale was explored by Wright et al. in 2024 [26] to develop a predictive model, termed the Inpatient Rehabilitation Fall (IRF) scale, by comparing the total FIM rating for assessing in-hospital falls among patients admitted for rehabilitation to the Morse scale. The IRF scale demonstrated acceptable accuracy in identifying patients who fell in the retrospective cohort of Wright’s study [26]. However, due to the retrospective nature of the study, these results cannot be used at a clinical level, and a further prospective assessment is required to confirm the validity of the IRF scale. In the present study, the motor FIM scale was not used as a tool for assessing the inclusion criteria, but as a tool for assessing outcome measures before discharge. However, from the results on the whole patient population (Table 2), we can speculate that other scales (e.g., Barthel Index, Table 4) should be considered to better target frail patients in our specific setting.

In the present study, the number of non-eligible patients for smart-belt application far exceeded that of eligible patients, underscoring the clinical and cognitive complexity of this population. Specific recommendations for older patients with cognitive impairment are scarce [5], but considerations in this regard are imperative, as deficits in executive functions are a known risk factor for older patients with neurological disorders. Notably, the highest percentage of falls occurred in very compromised patients not included in the study (13.9% vs. 4.2% among eligible patients), further emphasizing the importance of addressing the risk of falls in this vulnerable population. Indeed, the use of a smart belt for hip protection in this subgroup probably could not have contained the damage.

Moreover, only 14% of eligible patients accepted a smart belt to wear. In this small sample, despite some dropouts, the use of a smart belt was feasible and showed good adherence in 53% of cases, largely due to personal patient motivation to complete the study. No patients experienced significant serious events during the observation period. However, the only user who then refused to wear the belt and fell with minor consequences would have benefited from its use, as would the non-user patient who refused to participate in the study and fell with a hip fracture.

These findings align with those of Tarbert et al.’s study [30], which used the STEADI (Stopping Elderly Accidents and Death Initiative) criteria—based on the Minimum Data Set 3.0 algorithm—to identify patients at risk of falls across residential and institutional settings in USA. Tarbert et al.’s study involved a real-world case series of residents identified as being at high risk of major fall injuries within a long-term care setting, using a Tango belt connected with a mobile app [30]. Over nearly two years, 35 residents wore the smart belt, and six falls with airbag deployment occurred, coinciding with a reduction in the overall rate of falls with major injuries. Nevertheless, Tarbert et al. [30] demonstrated consistent patient adherence with the use of the Tango belt (80%), and although there was “no comparison between the effectiveness of smart belt intervention and the standard of care”, the number of falls was reduced by 60% and the device was implemented over a time span of 23 months in a total 35 residents. As we observed fall events mainly in patients who did not wear a smart belt, we cannot compare our data with the data of their study. 

### 4.2. Can We Effectively Identify Appropriate Patients to Wear Smart Belts? 

The incidence of falls among patients admitted to the rehabilitation units in the current study was in line with the institutional data from the same years. However, due to the limited number of available belts, the criteria for selecting patients were not sufficient to identify patients at real risk of falling. In fact, we lost a large proportion of patients, among whom 44 experienced falls. While there is currently no standard of care, Shany et al.’s review [24] suggests using wearable sensors for screening and assessing risk of falling in older people. Similarly, Ferreira’s 2022 review [11] updates the state-of-the-art knowledge in fall risk assessment systems, revealing that most studies rely on clinical scales. Recent studies have focused on gait speed estimated by using the 6 min walking test [31] or the Short Physical Performance Battery [32], which demonstrated clinical utility for fall-risk stratification to complement the STEADI guidelines over one and four years of follow-up among older adults, suggesting new possibilities for the identification of eligible patients. 

Successful implementation of smart belts depends not only on identifying eligible patients but also on providing useful recommendations for their use to ensure adherence. While current guidelines [5] strongly recommend the use of hip protectors in nursing homes, their use in rehabilitation settings, alongside digital and wearable technologies, is less explored in the literature. Previous Cochrane meta-analyses [33] indicated weak evidence for hip protectors’ efficacy in preventing fractures after a fall in long-term care facilities, highlighting the challenges in their daily implementation.

In addressing fall-related hip injuries in rehabilitation settings, both our study and Talbert et al.’s study [30] proposed solutions involving active wearables. However, the implementation of eligibility criteria for selecting patients remains limited to the use of a few specific scales, suggesting a need for developing and applying predictive models from in-hospital to home settings, as proposed by Wright et al. [26]. 

### 4.3. Is the Study Intervention Acceptable and Safe for Participants?

In our study, the acceptability of the smart belt among user patients remained a significant challenge, with only 35% completing the two-week observation period while wearing it. This adherence rate falls short of what Quigley et al. [34] observed in ambulatory older long-term care residents, where adherence reached 80%. However, their study involved less complex patients and a different recruitment setting.

As highlighted in Talbert et al.’s review [35], a major barrier to acceptance was discomfort, particularly the perception of the belt being too heavy, especially among women, and difficulty in putting it on and taking it off. Additionally, instances where the airbag was deployed accidentally led to patient agitation, resulting in their refusal to continue wearing the belt.

### 4.4. Does the Research Team Possess the Necessary Resources and Time to Manage the Study Intervention Effectively?

Similarly to the findings reported by Talbert et al. [30], our project faced challenges due to limited dedicated personnel in a busy rehabilitation environment. To address this, it may be beneficial to assign a dedicated nurse or physiotherapist to oversee the project, thereby freeing up the usual ward staff to continue their regular duties. This approach would allow for the integration and direct supervision of the new prevention program, which is based on the use of active wearable devices, within the existing rehabilitation program. Furthermore, it would enable the evaluation and potential expansion of the use of smart belts to other patient populations within our institution, such as cardiorespiratory patients, or to other facilities within the Maugeri Institute network.

### 4.5. Is It Possible to Modify Program Contents or Procedures to Make Them More Suitable?

Utilizing emerging technology borrowed from other industries presents an opportunity for implementing supplementary intervention in the older adult population at risk of falls and major fall injuries [30].

The first challenge in developing an effective intervention is to identify the population that would benefit the most from using the device. To date, there is no clinical consensus on which fall-prediction methods are effective in different clinical settings. A more innovative approach involves the use of machine-learning analysis to study fall prediction in older individuals who have not yet experienced falls, as demonstrated by Bargiotas [36]. Some authors have focused on the chronic stroke population, developing a pre-impact fall-detection model trained on data obtained from these high-risk patients [37], while others [31] have employed a similar approach in individuals with neurological disorders to predict a score measuring the individual capacity to maintain balance and gait in the presence of environmental demands. The study by Liuzzi et al. [31] on the use of data collected during short steady-state walking bouts (6MWT) through inertial measurement units and analyzed via machine learning is ongoing. A further point of consideration is the development of environmental sensors to collect data for identifying people at risk of falls. The modality of fall-injury protection remains to be determined.

## 5. Conclusions

Conducting feasibility research in the intervention research process is a determinant for accepting or discarding an intervention approach. It is clear that preventing falls in older people represents a complex challenge that requires a holistic and multidisciplinary approach. The use of innovative technologies, such as smart belts, offers promising opportunities to reduce the risk of falls and fall-related serious injuries. However, there are still significant challenges to overcome, such as adequate participant selection, acceptance of the proposed intervention, and identification of a user-friendly technology that is not cumbersome for participants.

The results of the current study confirm, as previously reported in the literature, that the criteria for participant selection may not be sufficient to effectively identify patients at risk of falls, highlighting the need for more precise selection criteria and new approaches for the fall-risk assessment. Additionally, both the acceptance and safety of the proposed intervention pose further challenges.

However, reviewing and adapting the participant selection criteria of existing intervention programs could help improve the effectiveness of the proposed intervention. Furthermore, the use of advanced technologies, such as machine learning, could enable the creation of more accurate predictive models to identify patients at risk of falls and personalize such a prevention intervention to be more effective.

## Figures and Tables

**Table 1:** Comparison between eligible (N = 118) and non-eligible (N = 317) patients upon admission (white columns). The gray columns present details on the comparison between two subgroups of eligible patients in italics: those who used the belt (users, N = 17) and those who refused the belt (non-users, N = 101).

Variables	Whole Population N = 435	Eligible (E)				Non-Eligible (NE)	
		Total N = 118	Users N = 17	Non-Users N = 101	Us vs. NUs *p*	Total N = 317	E vs. NE *p*
Age, years	77 (68–84)	77 (68–84)	*75 (62–79)*	*77 (69–84)*	*0.3338*	77 (68–84)	0.6678
Male, n (%)	170 (39)	38 (32)	*8 (47)*	*30 (30)*	*0.1702*	132 (42)	0.0924
BMI, Kg/m^2^	25 (22–28)	26 (23–29)	*25 (23–27)*	*26 (23–30)*	*0.2694*	25 (22–28)	0.0196
CIRS severity, score	1.9 (1.7–2.2)	1.8 (1.6–2.1)	*1.8 (1.6–1.9)*	*1.8 (1.7–2.1)*	*0.3300*	1.9 (1.7–2.2)	0.0187
LOS, days	34 (23–48)	30 (22–43)	*42 (21–53)*	*30 (22–42)*	*0.2466*	34 (23–50)	0.0520
Motor FIM, score	50 (31–62)	54 (38–67)	*64 (53–68)*	*51 (37–65)*	*0.0453*	47 (28–61)	0.0012
Barthel Index, score	54 (23–72)	66 (37–74)	*74 (65–77)*	*61 (31–74)*	*0.0186*	49 (20–70)	<0.0001
TUG *, score	23 (18–31)	24 (19–33)	*20 (16–21)*	*26 (20–35)*	*0.0034*	22 (18–31)	0.4454

Abbreviations: BMI, body mass index; CIRS, Cumulative Illness Rating Scale; sev, severity index; LOS, length of stay; FIM, Functional Independence Measure; TUG, Timed Up and Go Test; IC, informed consent; Us = users; NUs = non-users; E = eligible; NE = non-eligible; *p*, *p*-value for comparison between users and non-users (gray) and eligible (total) vs. non-eligible patients (total, white columns on the right side); * with about 50% of missing values.

**Table 2:** Comparison between fallers (N = 49) and non-fallers (N = 386) in the whole patient population (total admission), and comparison between fallers (N = 5) and non-fallers (N = 113) among eligible patients (N = 118, right side).

Variables	Whole Population N = 435	Whole Population (N = 435)			Eligible (N = 118)		
		Fallers N = 49	Non-Fallers N = 386	*p*	Fallers N = 5	Non-Fallers N = 113	*p*
Age, years	77 (68–84)	82 (77–84)	76 (67–83)	0.0005	84 (82–84)	76 (67–83)	0.0509
Male, n (%)	170 (39)	27 (55)	143 (37)	0.0223	3 (60)	35 (31)	0.3263
BMI, Kg/m^2^	25 (22–28)	25 (23–28)	25 (22–28)	0.6312	28 (23–30)	26 (23–29)	0.8082
CIRS sev, score	1.9 (1.7–2.2)	2.0 (1.8–2.2)	1.9 (1.7–2.2)	0.1094	2.1 (1.9–2.0)	1.8 (1.6–2.1)	0.1873
LOS, days	34 (23–48)	46 (34–63)	32 (22–47)	<0.0001	48 (36–65)	30 (22–43)	0.0450
Motor FIM, score	50 (31–62)	41 (26–53)	51 (32–63)	0.0499	60 (56–61)	54 (38–67)	0.6518
Barthel Index, score	54 (23–72)	41 (19–57)	56 (25–73)	0.0060	60 (31–61)	66 (38–75)	0.1849
TUG *, score	23 (18–31)	24 (17–33)	22 (18–31)	0.9820	20 (17–60)	24 (19–33)	0.7702

Abbreviations: BMI, body mass index; CIRS, Cumulative Illness Rating Scale; sev, severity index; LOS, length of stay; FIM, Functional Independence Measure; TUG, Timed Up and Go Test; * about 50% of missing values.

**Table 3:** Evaluation of the injuries that occurred in non-eligible patients and eligible patients who refused to use a smart belt.

	Fallers	Fall Consequences			With Hip Fracture
		No	Mild	Serious	
All	49	32	10	7	3
1. Non-eligible	44	29	9	6	2 **
2. Eligible	5	3	1	1	0
2a. Non-users	*4*	*3*	*0*	*1*	*1* *
2b. Users	*1*	*0*	*1*	*0*	*0*

Note: * This fall with hip fracture occurred in an eligible but non-user patient while slowly sliding out of the wheelchair. ** The other two falls occurred in non-eligible patients who removed their restraints from the bed/wheelchair.

**Table 4:** Pre-to-post comparison in users.

*Variable*	Pre-to-Post Comparison		
	Pre	Post	p
*Cognitive FIM, score*	33 (31–35)	33 (31–35)	0.9156
*Motor FIM, score*	64 (53–68)	76 (68–984)	0.0032
Total FIM, score	96 (79–102)	111 (97–14)	0.0072
*Tinetti Balance, score (n = 16)*	9 (7–13)	13 (11–14)	0.0334
*Tinetti Gait, score (n = 15)*	8 (7–10)	9 (8–10)	0.3868
Total Tinetti, score (n = 15)	16 (14–21)	21 (19–24)	0.0168
Barthel Index, score (n = 16)	74 (65–77)	90 (84–98)	<0.0001
TUG, score (n = 14)	20 (16–21)	18 (13–21)	0.4539

## Data Availability

Data are available from the corresponding author upon request.
